# Epidemiology, risk areas and macro determinants of gastric cancer: a study based on geospatial analysis

**DOI:** 10.1186/s12942-023-00356-1

**Published:** 2023-11-25

**Authors:** Binjie Huang, Jie Liu, Feifei Ding, Yumin Li

**Affiliations:** 1 Department of General Surgery, Second Hospital of Lanzhou University, Lanzhou, China; 2 Key Laboratory of the Digestive System Tumors of Gansu Province, Lanzhou, China; 3 Lanzhou University, Lanzhou, China

**Keywords:** Gastric cancer, Epidemiology, Social and environmental factors, Risk areas, Spatial analysis

## Abstract

**Background:**

Both incidence and mortality of gastric cancer in Gansu rank first in china, this study aimed to describe the recent prevalence of gastric cancer and explore the social and environmental determinants of gastric cancer in Gansu Province.

**Methods:**

The incidence of gastric cancer in each city of Gansu Province was calculated by utilizing clinical data from patients with gastric cancer (2013–2021) sourced from the medical big data platform of the Gansu Province Health Commission, and demographic data provided by the Gansu Province Bureau of Statistics. Subsequently, we conducted joinpoint regression analysis, spatial auto-correlation analysis, space–time scanning analysis, as well as an exploration into the correlation between social and environmental factors and GC incidence in Gansu Province with Joinpoint_5.0, ArcGIS_10.8, GeoDa, SaTScan^TM^_10.1.1 and GeoDetector_2018.

**Results:**

A total of 75,522 cases of gastric cancer were included in this study. Our findings suggested a significant upward trend in the incidence of gastric cancer over the past nine years. Notably, Wuwei, Zhangye and Jinchang had the highest incidence rates while Longnan, Qingyang and Jiayuguan had the lowest. In spatial analysis, we have identified significant high-high cluster areas and delineated two high-risk regions as well as one low-risk region for gastric cancer in Gansu. Furthermore, our findings suggested that several social and environmental determinants such as medical resource allocation, regional economic development and climate conditions exerted significant influence on the incidence of gastric cancer.

**Conclusions:**

Gastric cancer remains an enormous threat to people in Gansu Province, the significant risk areas, social and environmental determinants were observed in this study, which may improve our understanding of gastric cancer epidemiology and help guide public health interventions in Gansu Province.

## Background

Cancer remains a significant global health threat, with gastric cancer ranking as the fifth most common in incidence and fourth in tumor-related deaths among all cancers. Recent data on cancer indicates that there were one million new cases of gastric cancer and 76,900 gastric cancer related deaths in 2020, with a male-to-female gender ratio of approximately 2:1 [1]. In recent decades, there has been a global decline in the incidence and mortality of gastric cancer due to the implementation of various tumor screening methods and anti-cancer strategies. From 1990 to 2019, the global age-standardized incidence (ASI) of gastric cancer decreased from 22.44/100,000 to 15.59/100,000, and the age-standardized mortality (ASM) decreased from 20.48/100,000 to 11.88/100,000; meanwhile, the ASI of GC in china decreased from 37.56/100,000 to 30.64/100,000, and the ASM decreased from 37.73/100,000 to 21.72/ 100,000 [2]. Although there is a steady decline, the incidence of gastric cancer in China remains significantly higher than that of other countries, particularly in Gansu province located in the northwest region; both the incidence and mortality of gastric cancer are relatively high throughout China.

Research has demonstrated that both genetic susceptibility and environmental factors contribute to the development of cancer [3–5]. In addition to traditional genetic factors, environmental factors such as dietary habits, lifestyle choices, environmental pollution (air, soil and water pollution), regional climate (rainfall, ambient temperature, humidity and sunshine exposure), as well as various geographical features such as altitude and slope also have an impact on cancer [6–8]. Spatial statistics is an interdisciplinary subject composed of geography and statistics, it has been widely used in many fields, including geography, epidemiology, economics and disease surveillance in recent years [9–11]. The field of spatial statistics distinguishes itself from traditional statistics by employing spatial auto-regression analysis and hot spot analysis to assess the association and cluster classification between different entities. Additionally, it utilizes spatial scanning techniques to identify distinct risk clusters. In this study, we used traditional statistics and spatial statistics to describe the epidemiologic characteristics of gastric cancer in Gansu. Meanwhile, Gansu's elongated and narrow shape contributes to significant variations in regional climate, geographic features, and socioeconomic factors; in order to investigate the social and environmental determinants of gastric cancer in Gansu province, we employed Geodetector as a tool to unveil the intricate interactions between different macro factors and gastric cancer.

## Methods

### Data sources

All patient data were retrieved from the medical big data platform of the Gansu Province Health Commission. Demographic, medical resource allocation, and economic data from the Gansu Province Bureau of Statistics. Topography, agrotype, and vegetational form data for Gansu province were extracted by masking from corresponding national datasets obtained from the Resource and Environment Science and Data Center. Altitude and slope data were sourced from Geo-spatial Data Cloud. Regional rainfall, ambient temperature, humidity, sunshine duration and diurnal temperature data were derived from global climatic datasets available at High-resolution gridded datasets.

### Statistical analysis

The patient data were collected from 266 hospitals across Gansu province over a span of nine years (2013–2021), encompassing patients' gender, age, and address information while ensuring the concealment of their name and ID number details. We determined the precise residential city and region (rural or urban) of each patient based on their address information, enabling us to calculate the gastric cancer incidence for every city in Gansu province. Additionally, we employed Joinpoint regression analysis to analyze temporal trends of the gastric cancer incidence in Gansu.

In the Geodetector analysis, we employed the q statistic to quantitatively assess the influential power of a specific independent variable on the dependent variable. The q value represents the extent to which the independent variable can comprehensively explain the variability in the dependent variable. A q value of 1 indicates that the independent variable can comprehensively account for the variation in the dependent variable, whereas a value of 0 indicates no discernible contribution from the independent variable. The calculation of the q value can be performed using the following formula.

$${\text{q}} = 1 - \frac{{\sum\limits_{h = 1}^{L} {N_{h} \sigma_{h}^{2} } }}{{N\sigma^{2} }}$$

In the equation, N represents the total area of the study region and σ^2^ denotes the variance of disease incidence. The incidence is dispersed to the L strata (h = 1, 2, …L). N_h_ and $\sigma_{h}^{2}$ represent stratum h and its variance. Statistical significance was set at p < 0.05.

The analysis of interactive effects determines whether two distinct independent factors have a combined impact on the dependent variable. We identified the interactive effect of two different independent factors on the dependent variable by calculating their interactive q value. The detailed interactive effect can be categorized into five types as listed below:

**Table:** 

Interactive type	Interactive effect
q(X1 ∩ X2) < Min(q(X1), q(X2)) Min(q(X1), q(X2)) < q(X1 ∩ X2) < Max(q(X1), q(X2)) q(X1 ∩ X2) > Max(q(X1), q(X2)) q(X1 ∩ X2) = q(X1) + q(X2) q(X1 ∩ X2) > q(X1) + q(X2)	Non-linearity reduction Univariate non-linearity reduction Bivariate enhancement Independence Non-linearity enhancement

The Poisson model implemented in SaTScan 10.1.1 software was utilized to identify areas of high or low risk for gastric cancer incidence in Gansu province. Detailed information on how the spatial scan statistic within SaTScan identifies cancer clusters can be found at http://www.satscan.org. Additionally, the map displaying gastric cancer incidence rates for each city in Gansu province and all processes prior to Geodetector were processed using ArcGIS 10.8.

## Results

### The prevalence of gastric cancer in Gansu

#### Age

**Fig. 1:**
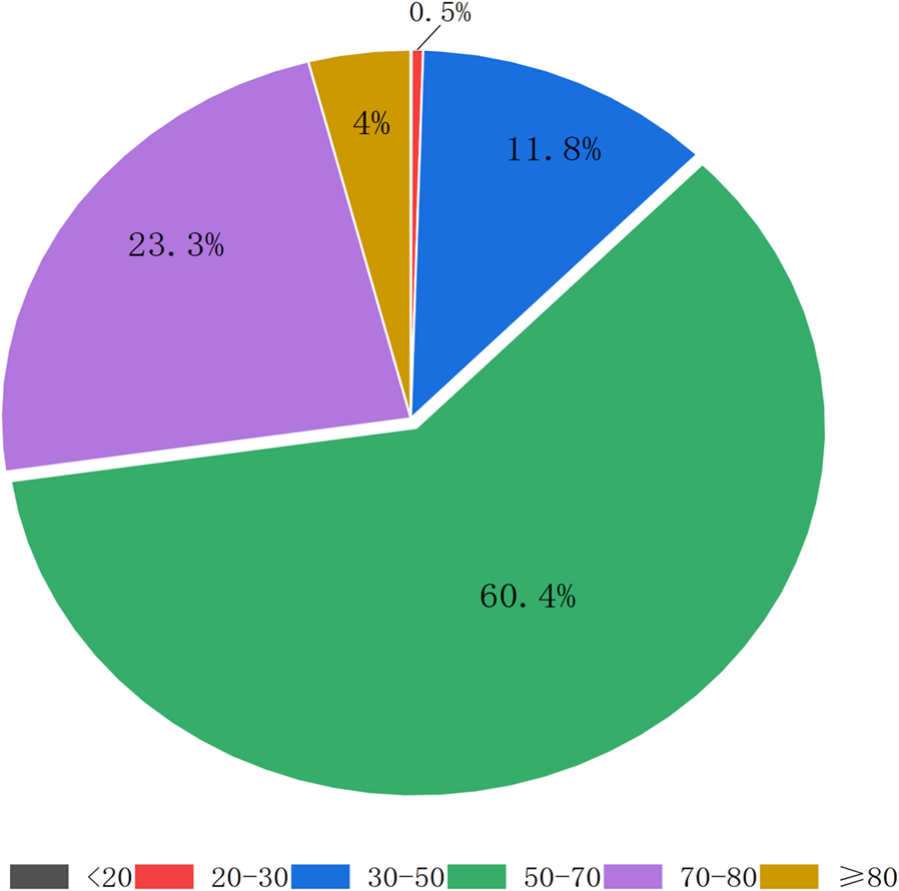
The age composition of patients included

**Fig. 2:**
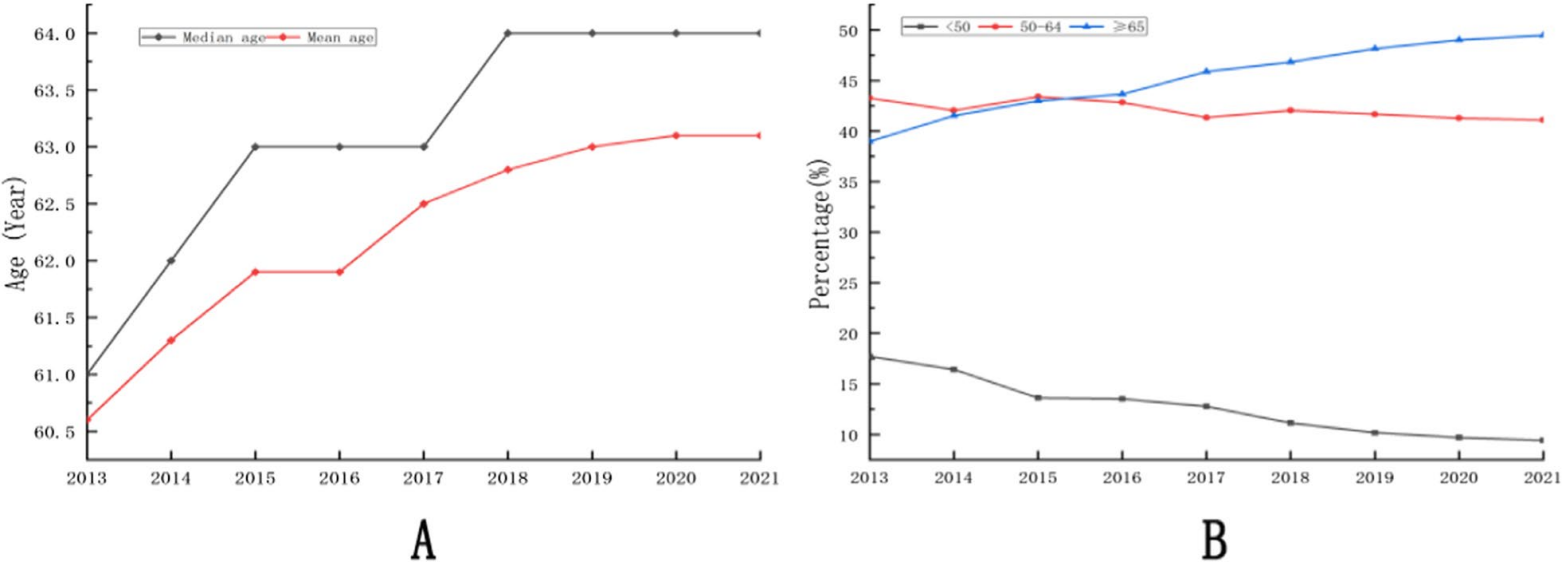
The mean age, median age and the trend of age proportion of patients included (**A**, mean age and median age. **B**, the trend of age proportion.)

A total of 75,522 gastric cancer patients with complete age information were ultimately included in our study. The mean age was 62.4 ± 10.8 years old and the median age was 63 years old, with most falling into the middle-aged to elderly range (50–70 years old). Fig. 1 displays the age distribution of these patients. As shown in Fig. 2, both mean and median ages varied across different years and exhibited an increasing trend from 2013 to 2021. In addition, we found that the proportion of gastric cancer patients whose age was younger than 50 years old and older than 65 years old increased, and the proportion of gastric cancer patients whose age ranged between 50 and 64 decreased in recent years.

#### Gender

**Fig. 3:**
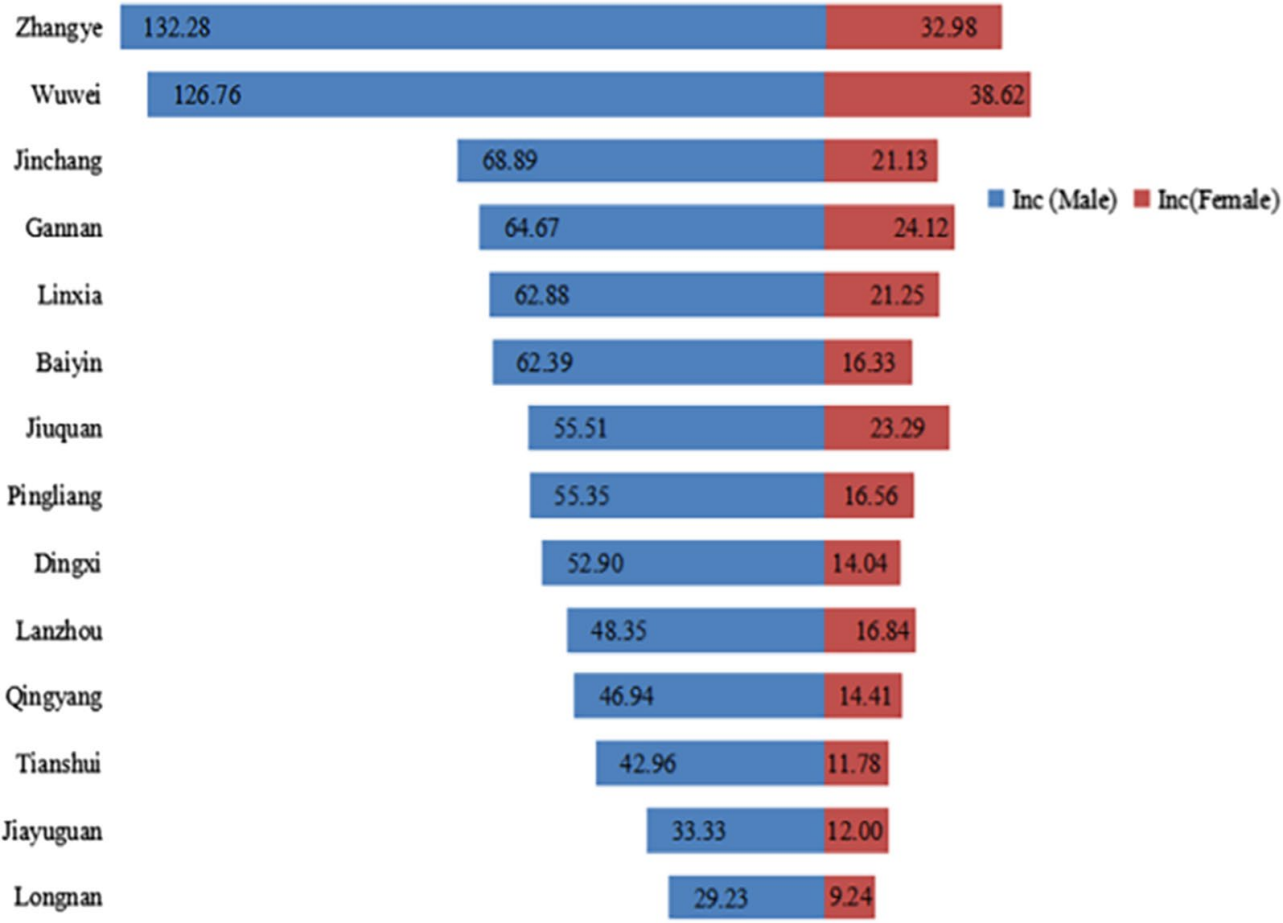
The gastric cancer incidence for different gender in different cities (1/100,000)

A total of 74,906 patients with complete gender information were included in our study, comprising 57,761 males and 17,145 females at a male-to-female ratio of 3.4:1.0. According to the latest Chinese census data, we found that the city with the highest incidence for males was Zhangye while Wuwei had the highest incidence for females; Longnan had the lowest incidence for both genders (Fig. 3).

#### Rural and urban regions

**Fig. 4:**
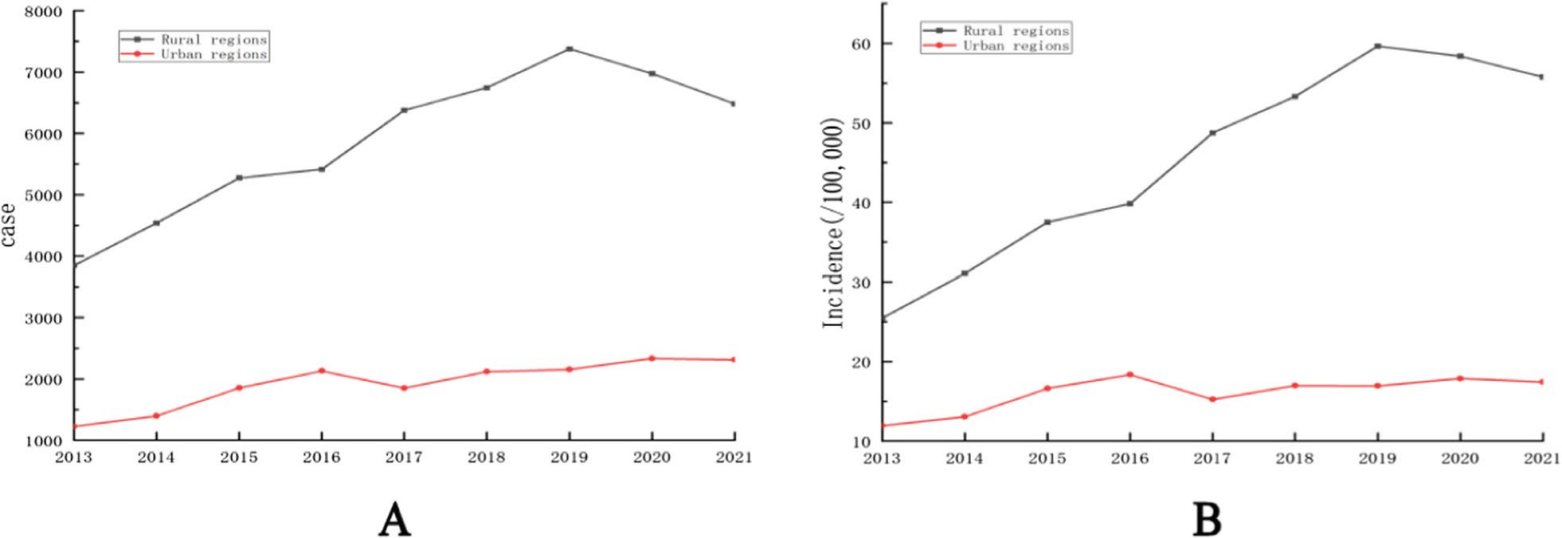
The cases and incidence of gastric cancer in rural and urban regions(**A**, cases. **B**, incidence.)

A total of 70,405 gastric cancer patients with complete residential address information were included in this study, and the rural–urban ratio of cases was 3.1:1.0. As illustrated in Fig. 4, both the incidence and prevalence of gastric cancer were significantly higher in rural regions than urban regions during recent years.

#### GC incidence of gansu province

**Fig. 5:**
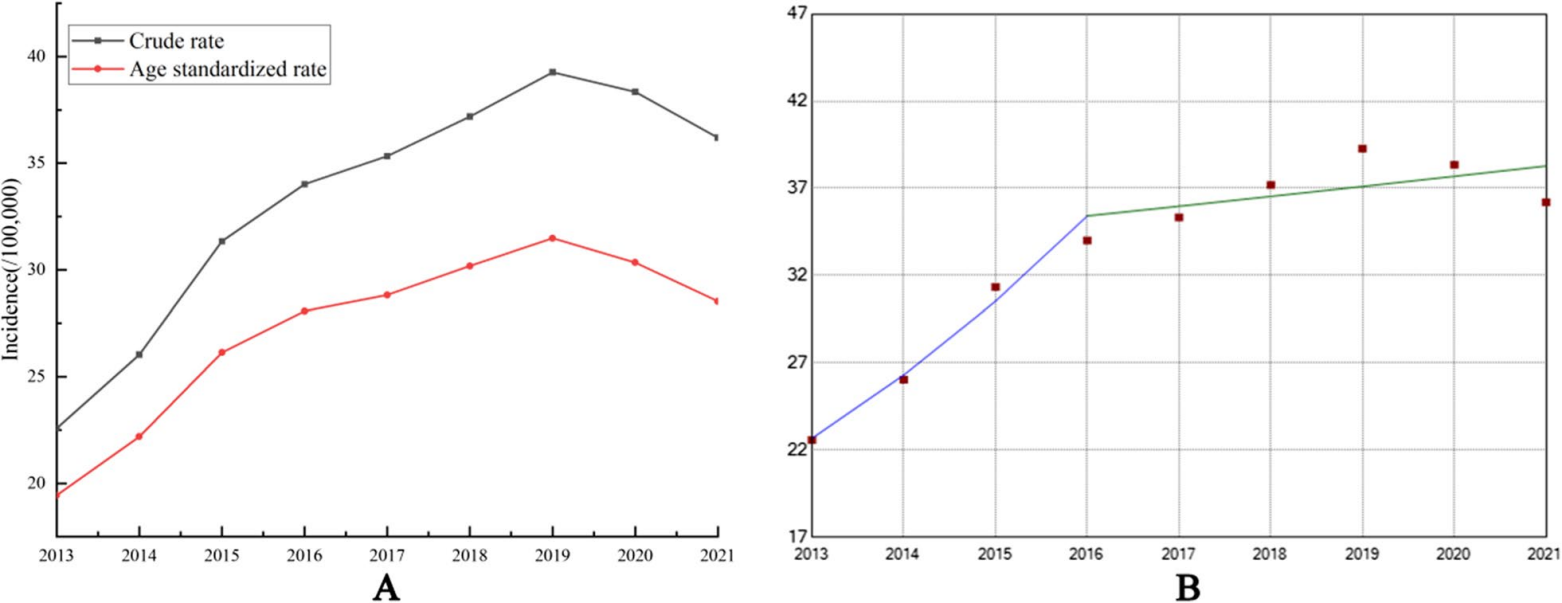
Time trends of incidences of gastric cancer in Gansu province from 2013 to 2021 (**A**, Incidence. **B**, Joinpoint regression for ASR.)

As shown in Fig. 5. Our analysis revealed a significant upward trend in the crude incidence rate and age standardized rate (ASR) of gastric cancer in Gansu province between 2013 and 2021, with an AAPC of 5.55% (95% CI 5.05, 9.01; p < 0.05). Joinpoint regression identified two distinct time segments with different APC values during this time frame. In segment 1 (2013–2016), the ASR showed a significant increase of 16.02% per year (APC = 14.31; 95% CI 8.33, 29.70; p < 0.05), while in segment 2 (2016–2021), it increased by only 0.61% per year, which was not statistically significant (APC = 0.61; 95% CI −4.42, 3.03; p > 0.05).

### Regional distribution of gastric cancer patients included

**Fig. 6:**
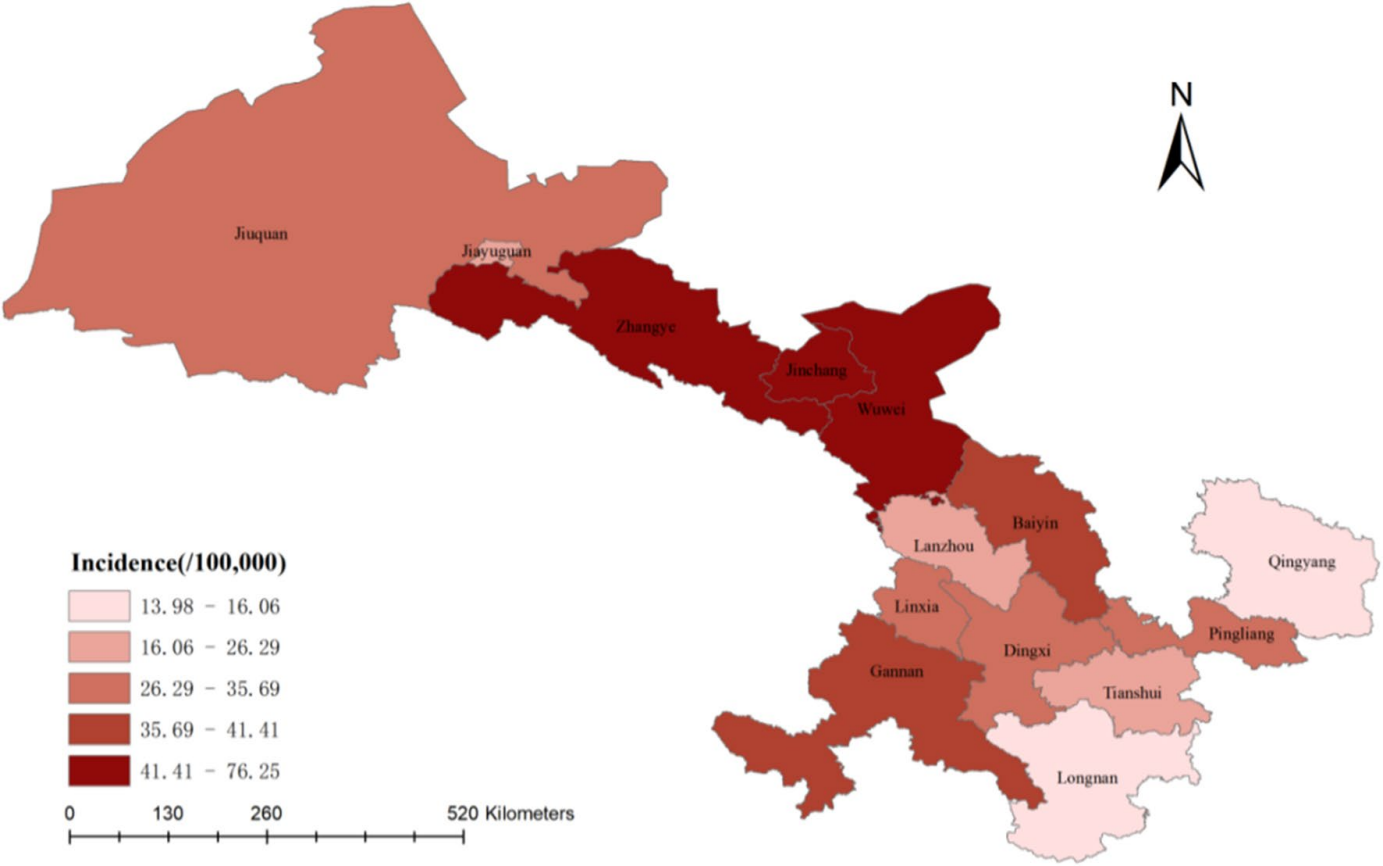
The prevalence map of gastric cancer in Gansu

As presented in Table 1 and Fig. 6, Wuwei, Lanzhou, and Zhangye were identified as the top three cities with the highest cumulative number of gastric cancer patients in Gansu Province; whereas Jiayuguan, Gannan, and Jinchang were recognized as the last three cities with the smallest cumulative number of gastric cancer patients. The top three cities with the highest incidence were Wuwei, Zhangye and Jinchang; and the last three cities with the lowest incidence were Longnan, Qingyang and Jiayuguan.

**Table 1:** Regional distribution of gastric cancer patients included in Gansu

Region	Cases	Population	Incidence(/100,000)	ASR(/100,000)
Wuwei	1197	1,569,800	76.25	58.80
Zhangye	788	1,150,600	68.49	50.23
Jinchang	289	445,500	64.87	47.78
Gannan	286	690,600	41.41	42.53
Baiyin	633	1,570,800	40.30	30.90
Linxia	735	2,059,300	35.69	35.05
Jiuquan	380	1,067,300	35.60	27.51
Pingliang	651	1,912,700	34.04	26.18
Dingxi	831	2,574,800	32.27	25.98
Lanzhou	1086	4,130,700	26.29	21.67
Tianshui	701	3,065,900	22.86	19.54
Jiayuguan	60	287,900	20.84	16.76
Qingyang	351	2,186,200	16.06	12.46
Longnan	343	2,453,200	13.98	12.17

Cases, the average number of gastric cancer patients; Population, the average resident population of every city in Gansu; Incidence, the crude rate of gastric cancer of every city in Gansu

### Spatial epidemiological analysis of the gastric cancer in Gansu

#### Spatial analysis (SA)

**Fig. 7:**
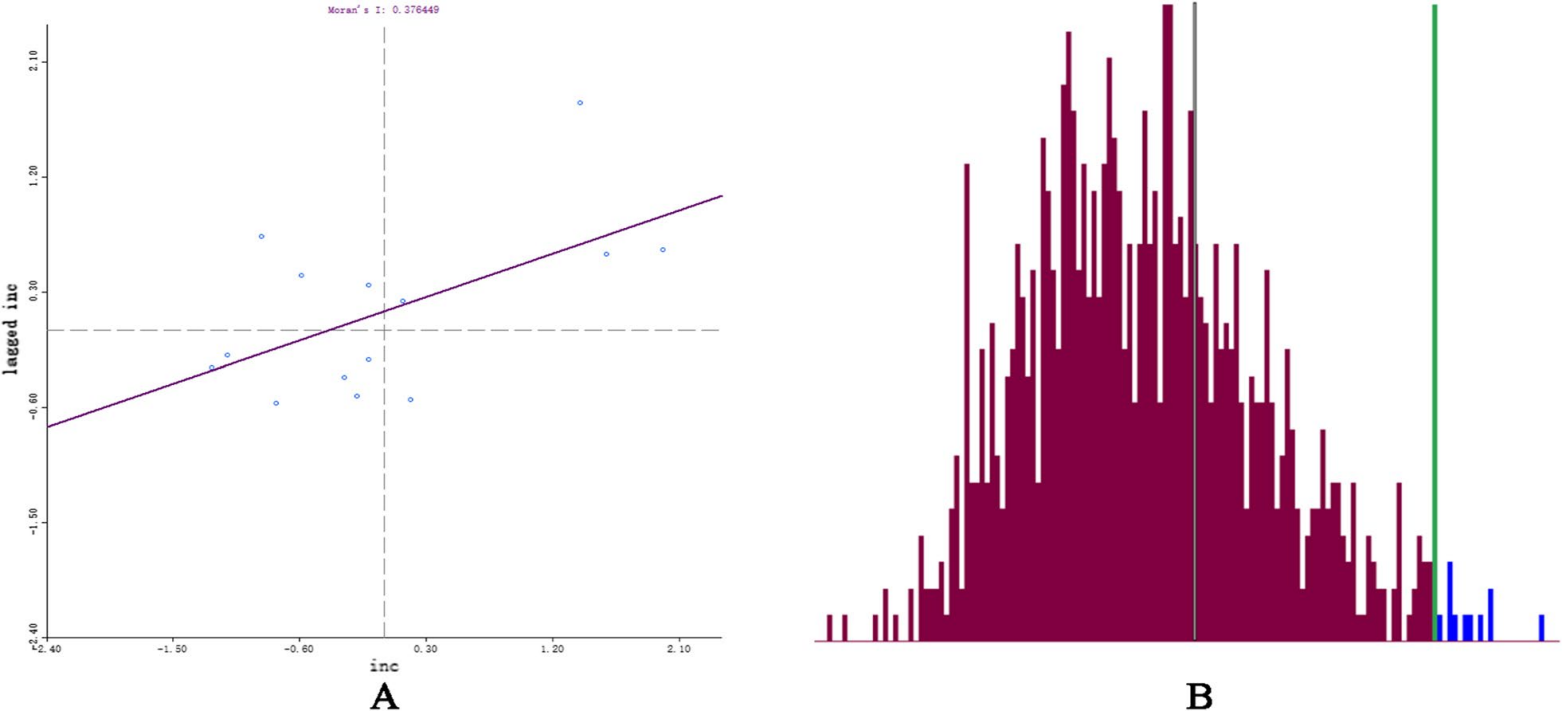
Global auto-correlation analyses (**A**, Moran`s index scatter plot. **B**, Permutation test.)

**Fig. 8:**
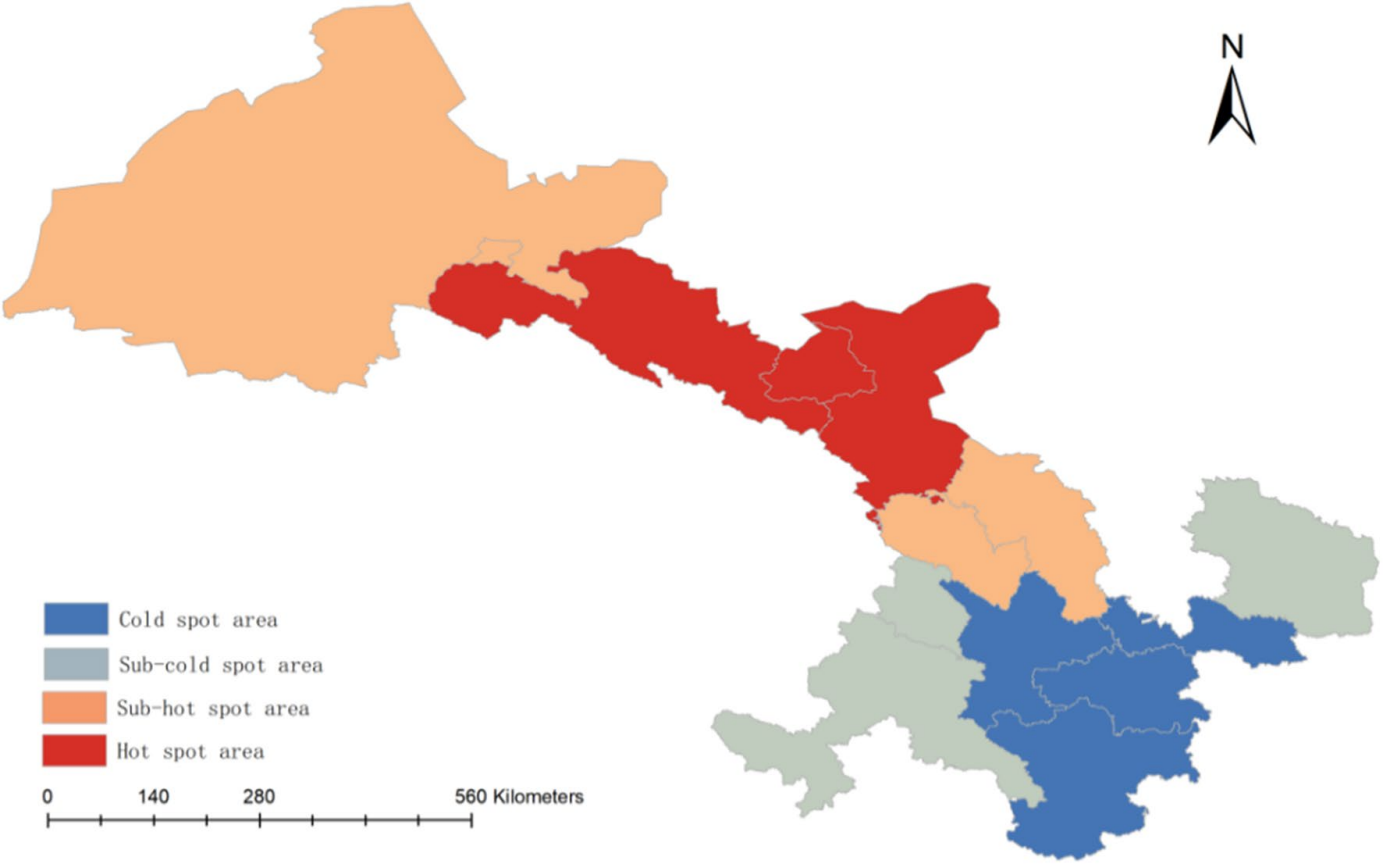
Hot spot analysis of gastric cancer incidence in Gansu province

**Fig. 9:**
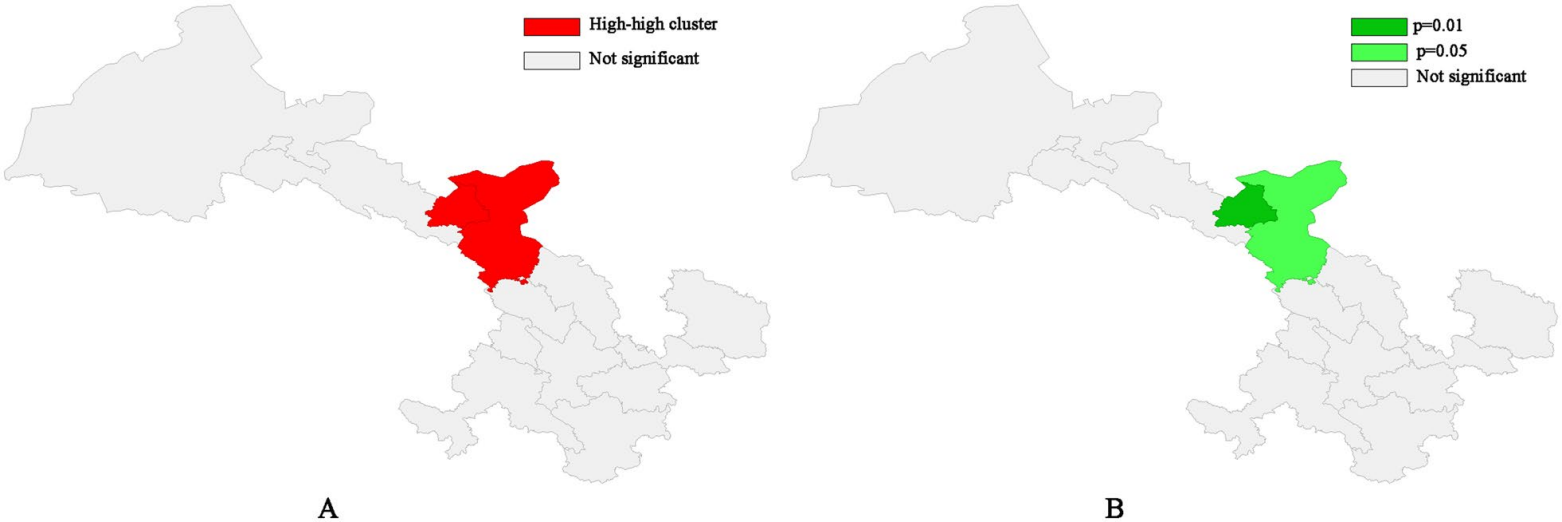
Local auto-correlation analysis (**A**, Clustering map. **B**, Significance map)

We conducted both global and local auto-correlation analyses, revealing a clustered pattern of gastric cancer incidence in Gansu Province based on the global Moran's index (Moran's index = 0.38, Z = 2.46, p < 0.05, Fig. 7). We further completed cold-hot spot analysis and found that the spatial distribution of gastric cancer in Gansu province exhibited significant regularity, as shown in Fig. 8, the northern region of Gansu province represents a central hots pot area with higher incidence rates, while the southern region is characterized by lower incidence rates and serves as a leading cold spot area. Based on local Moran's index calculations, we identified a significant high-high cluster area in Wuwei and Jinchang; however, no significant low-low cluster areas were observed throughout Gansu province (Fig. 9).

#### Spatial scanning analysis based on SaTScan™

**Fig. 10:**
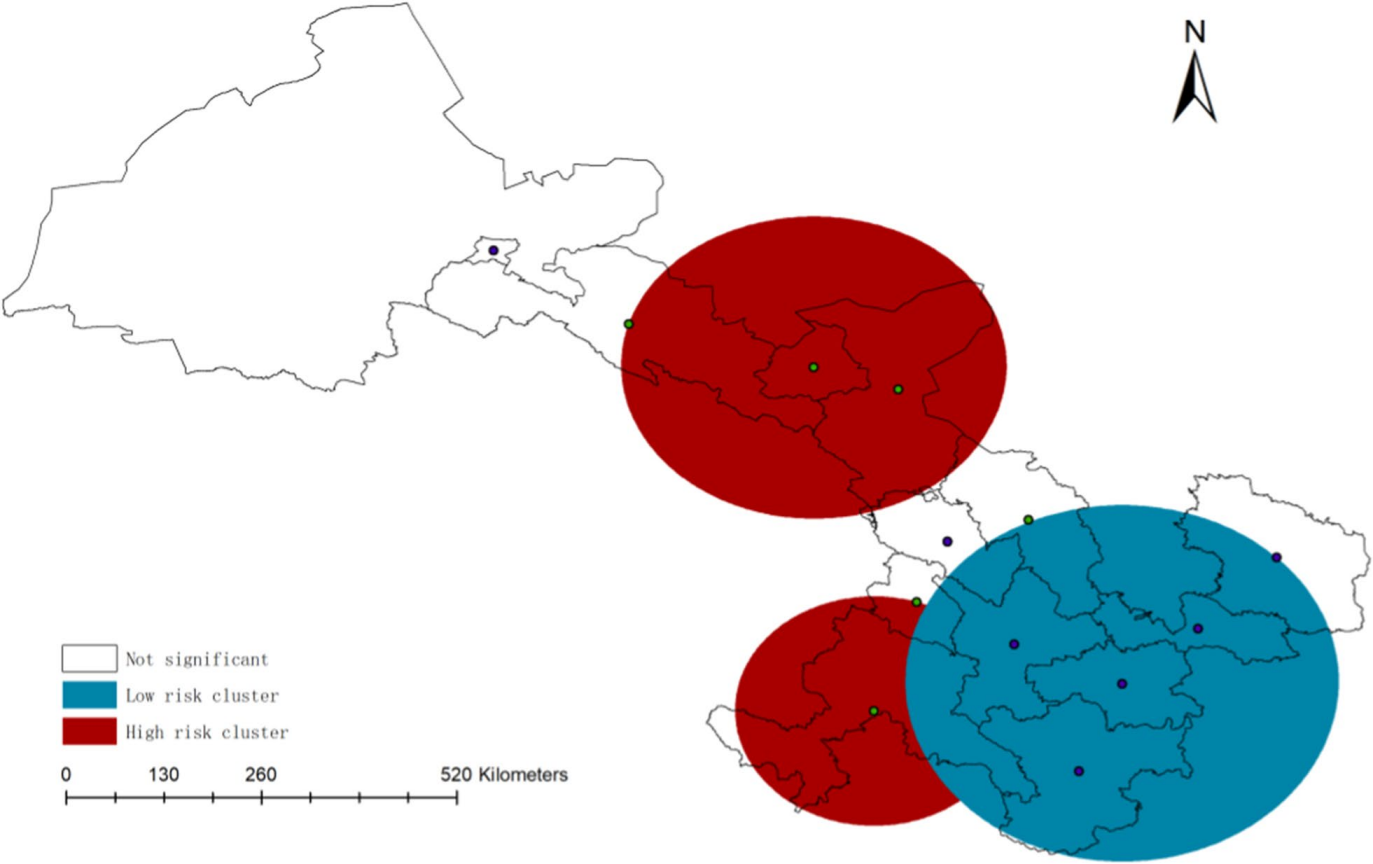
Purely spatial clusters map of GC in Gansu province

We used SaTScan^™^ software to identify significant spatial clusters of gastric cancer in Gansu. As shown in Table 2 and Fig. 10, two high risk clusters and one low risk cluster were observed in the purely spatial analysis.

**Table 2:** Gastric cancer cluster details based on purely spatial analysis

Type	Cluster	Cities	Observed cases	Expected cases	RR	p value
High	1 2	Jinchang, Wuwei, Zhangye Linxia, Gannan	20,473 9189	9493 8195	2.61 1.14	< 0.01 < 0.01
Low	1	Longnan, Tianshui, Dingxi, Pingliang, Qingyang	25,897	36,333	0.56	< 0.01

The most likely high risk cluster was found in the north of Gansu province, Wuwei, Zhangye and Jinchang were included in this area. In this area, there were 20,473 observed cases of gastric cancer and 9434 expected cases, with a statistically significant 161% increased risk of GC (RR = 2.61, p < 0.01). Another secondary high risk cluster was found in the southwest of Gansu province, Linxia and Gannan were included in this area. There were a total of 9189 observed cases of GC and 8195 expected cases, with a RR of 1.14 (p < 0.01), which implied that there was a 14% increased risk in this area compared with the total population in Gansu province. The most likely low risk cluster was found in the south of Gansu, Longnan, Tianshui, Dingxi, Pingliang and Qingyang were included in this area, there were 25,897 observed cases of GC and 36,333 expected cases, with a RR of 0.56, implying that, there is a statistically significant 0.44% decreased risk of GC in this area.

We further complete space–time scanning with the data. As shown in Table 3, the high risk and low risk clusters identified through space–time analysis closely resemble those found in purely spatial clustering.

**Table 3:** Gastric cancer cluster details based on space–time analysis

Type	Cluster	Years	Cities	Observed cases	Expected cases	RR	p value
High	1 2	2016–2019 2018–2021	Jinchang, Wuwei, Zhangye Linxia, Gannan, Dingxi	10,038 8411	4166 7054	2.63 1.22	< 0.01 < 0.01
Low	1	2013–2016	Longnan, Tianshui, Dingxi, Pingliang, Qingyang	8773	16,417	0.47	< 0.01

### Macro determinants of gastric cancer based on Geodetector

#### Social economic data of every city in Gansu

As presented in Table 4, we calculated the average values of five key economic indicators and three variables related to allocation of medical source in every city of Gansu province, in which, the units of five economic variables are 10,000 yuan.

**Table 4:** The mean values of multiple economic indicators across all cities in Gansu

City	A	B	C	D	E	F	G	H
Lanzhou	2386.91	50.94	863.92	1472.05	6.3	6	68	83
Jiayuguan	234.36	4.22	143.73	86.41	9.27	4	74	98
Jinchang	265.64	20.02	161.37	84.24	5.74	12	62	83
Baiyin	466.64	68.82	198.95	198.88	2.76	9	52	58
Tianshui	584.00	98.87	180.08	305.05	1.78	12	50	47
Wuwei	447.59	114.72	134.78	198.1	2.54	12	69	67
Zhangye	396.46	97.42	102.54	196.5	3.28	13	77	74
Pingliang	386.15	89.91	112.53	183.71	1.87	14	67	61
Jiuquan	599.98	81.30	244.38	274.30	5.41	9	63	73
Qingyang	659.10	79.08	341.8	238.22	2.95	9	47	48
Dingxi	339.96	74.51	71.00	194.45	1.24	11	60	44
Longnan	351.60	70.56	80.60	200.44	1.37	20	44	45
Linxia	241.77	37.48	49.76	154.54	1.19	9	50	38
Gannan	153.83	32.75	25.47	95.61	2.18	13	41	60

A, GDP; B, Output value of first industry; C, Output value of secondary industry; D, Output value of third industry; E, GDP per capita; F, Number of medical institutions per 10,000 people; G, Number of hospital beds per 10,000 people; H, Number of health technical staffs per 10,000 people

#### Natural environmental data of every city in Gansu

**Fig. 11:**
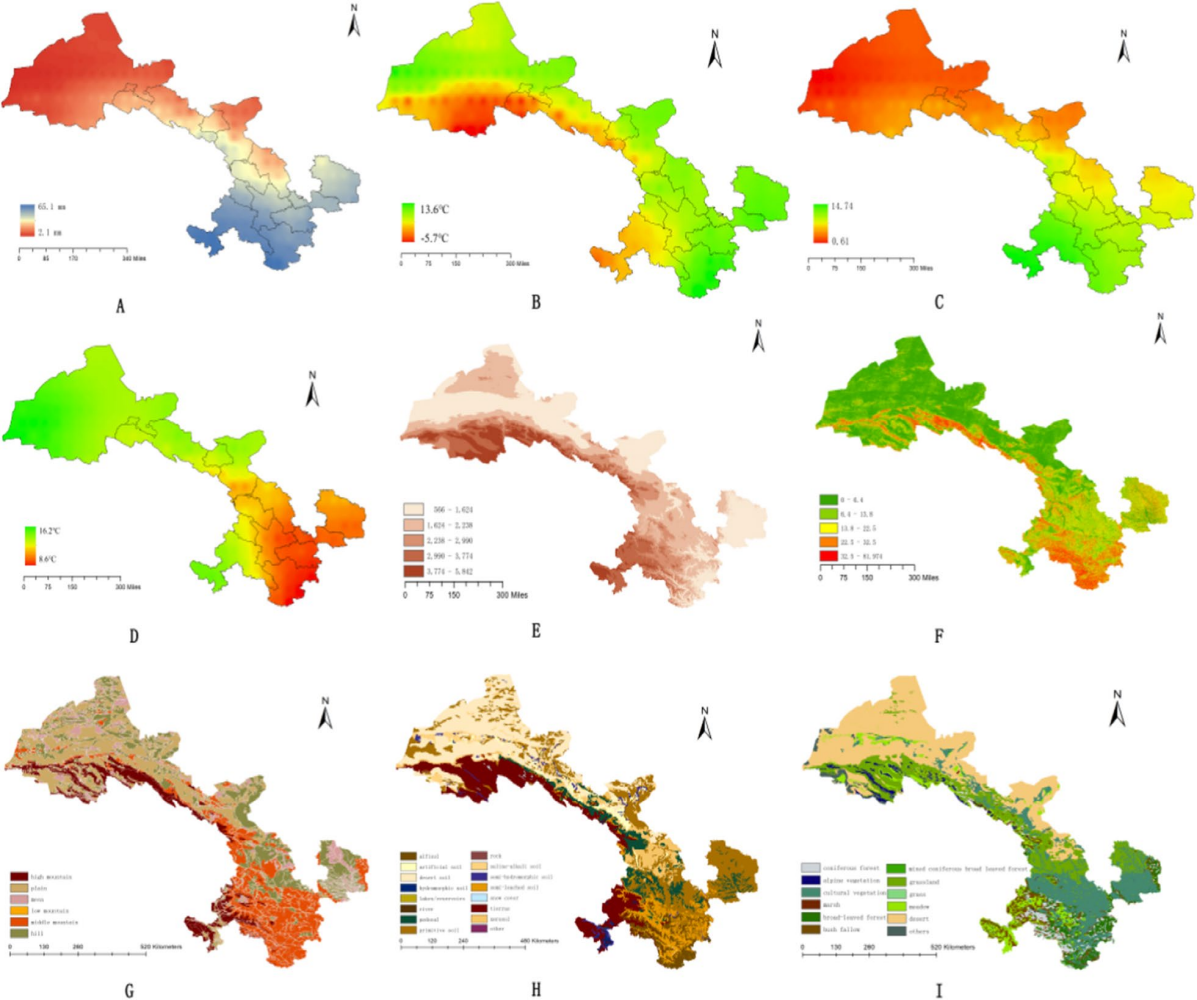
Natural environmental data of Gansu province. (**A**, regional rainfall. **B**, ambient temperature data. **C**, ambient humidity data. **D**, diurnal temperature variation data. **E**, altitude data. **F**, slope data. **G**, topography data. **H**, agrotype data. **I**, vegetational form data.)

In the study, we obtained the topography data, agrotype data, vegetational form data, altitude data, slope data, regional rainfall data, ambient temperature data, ambient humidity data and diurnal temperature data of Gansu province; these data were all extracted by mask from Chinese or global data which were downloaded from some official public database, including High-resolution gridded datasets, Geospatial Data Cloud and Resource and Environment Science and Data Center. The results are shown in Fig. 11.

#### Factor detector result of Geodetector

According to the aforementioned social-economic, medical source allocation and natural environmental data, we run Geodetector to explore the correlation between gastric cancer incidence and these factors. As shown in Table 5, all included factors exhibited statistically significant determinant power on gastric cancer in Gansu province (p < 0.05). Furthermore, we assessed the influential power of each factor and discovered that all except altitude, slope, and topography (q < 0.1) significantly impacted gastric cancer incidence. Among the remaining factors, three medical resource allocation variables, three economic indicators (Output value of second industry, GDP per capita and GDP), and diurnal temperature variation had a greater determinant power than others. Notably, the number of health technical staff per 10,000 people was the most influential factor that determined the gastric cancer distribution in Gansu province (q = 0.898). Except for these seven main factors, other factors such as the output value of the primary industry, regional rainfall, ambient humidity, output value of the tertiary industry, ambient temperature, agrotype and vegetational form also have a significant impact on gastric cancer distribution. However, their influence is all less than 50%.

**Table 5:** The factor detector result of Geodetector

Factor	q value	P value
Number of health technical staffs per 10,000 people	0.898	< 0.01
Output value of second industry	0.783	< 0.01
GDP per capita	0.725	< 0.01
Number of hospital beds per 10,000 people	0.654	< 0.01
GDP	0.638	< 0.01
Number of medical institutions per 10,000 people	0.536	< 0.01
Diurnal temperature variation	0.512	< 0.01
Output value of first industry	0.428	< 0.01
Regional rainfall	0.413	< 0.01
Ambient humidity	0.395	< 0.01
Output value of third industry	0.372	< 0.01
ambient temperature	0.303	< 0.01
Agrotype	0.238	< 0.01
Vegetational form	0.206	< 0.01
Slope	0.085	< 0.01
Altitude	0.080	< 0.01
Topography	0.062	< 0.01

#### Interactive detector result of Geodetector

**Fig. 12:**
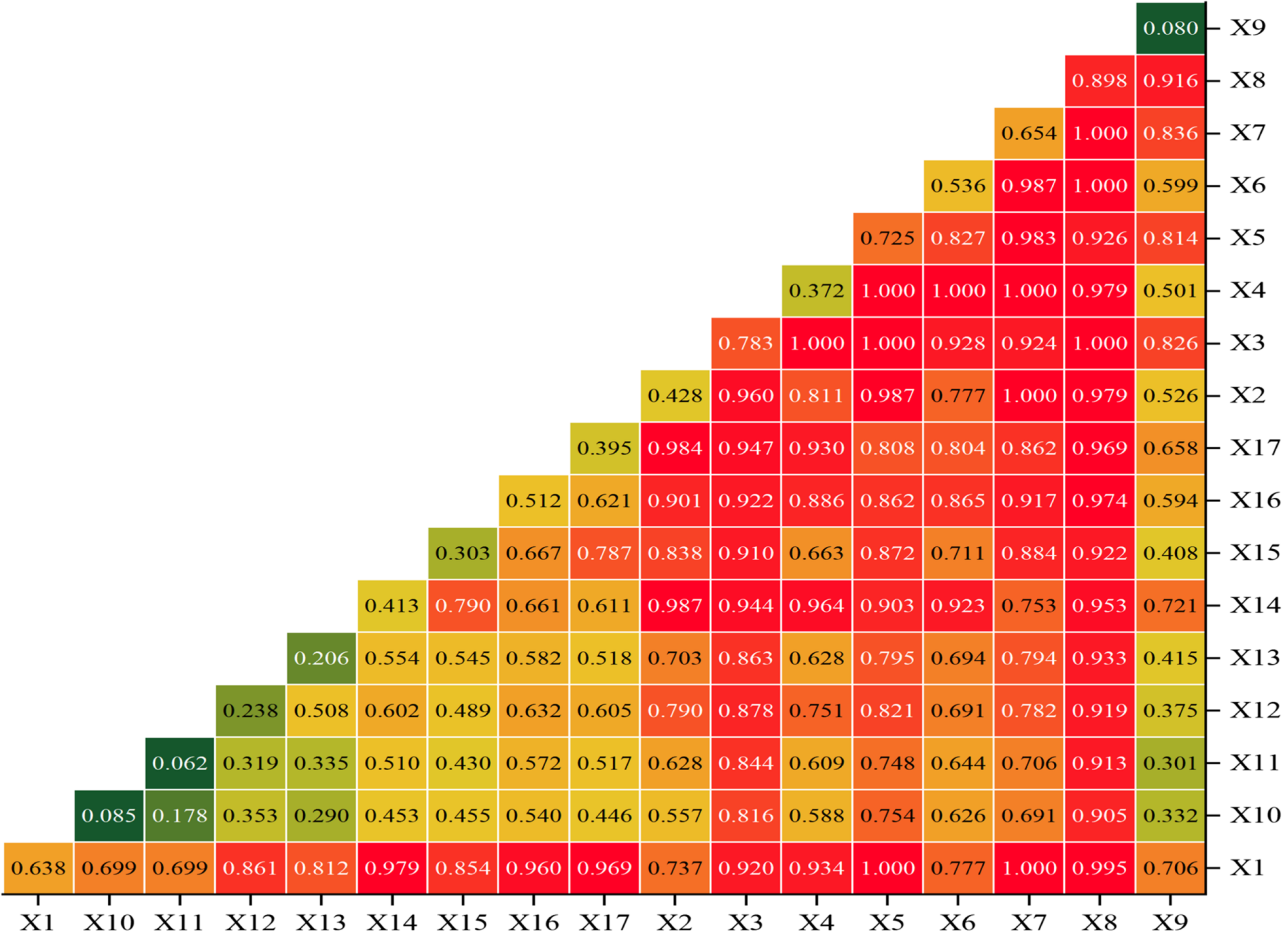
Interactive impact of risk factors on gastric cancer incidence in Gansu (X1, GDP; X2, Output value of first industry; X3, Output value of second industry; X4, Output value of third industry; X5, GDP per capita; X6, Number of medical institutions per 10,000 people; X7, Number of hospital beds per 10,000 people; X8, Number of health technical staffs per 10,000 people; X9, Altitude; X10, Slope; X11, Topography; X12, Agrotype; X13, Vegetational form; X14, Regional rainfall; X15, Ambient temperature; X16, Diurnal temperature variation; X17, Ambient humidity)

In this study, we used the interactive detector to find out whether the two risk factors included acted separately or synergistically. As shown in Fig. 12, our findings indicated that any combination of paired risk factors could significantly amplify their impact on gastric cancer in Gansu province through various forms of interaction, including bivariate enhancement and non-linearity enhancement. We used the interaction of ambient temperature and GDP per capita, regional rainfall and altitude as examples to explain the interactive effect of two different risk factors. According to Fig. 12, we could find the independent influential power of these two factors; their q values were 0.413 and 0.725 respectively; The independent determinant power of regional rainfall was found to be significantly smaller than that of GDP per capita. However, the combinational influential power of these two factors exhibited a synergistic effect with a q value of 0.903, which surpassed their individual q values but fell short of their sum and thus suggesting a bivariate enhancement effect on gastric cancer in Gansu province. Meanwhile, the combination of regional precipitation and tertiary industry output significantly enhances their independent determinant power on the dependent variable with a value of 0.964, surpassing the significance of either individual q value (0.413 and 0.372); moreover, it exceeds their sum (sum(q) = 0.785), indicating a non-linear enhancement on gastric cancer in Gansu province.

## Discussion

The latest global cancer statistics have revealed a consistent decline in the incidence of gastric cancer, particularly non-cardiac gastric cancer, worldwide. Additionally, it has been observed that the male-to-female gender ratio stands at 2:1.[1, 2]. However, we have observed a rising trend and a higher gender ratio for gastric cancer in Gansu, indicating that gastric cancer continues to pose a significant threat to the local population, particularly among males residing in rural regions of Wuwei, Zhangye, and Jinchang. These three cities have been identified as having the highest incidence of gastric cancer in Gansu. Meanwhile, the spatial epidemiological analysis revealed a significant regional disparity in the distribution of gastric cancer in Gansu, with distinct risk clusters observed along both north–south and west–east directions. Given the considerable variations in regional climate, geographic features, and socioeconomic status resulting from Gansu's elongated and narrow shape, it is plausible that there exists a potential correlation between these macro factors and gastric cancer incidence in Gansu.

In the Geodetector analysis, we observed a significant influence of social economic status (SES) on the incidence of gastric cancer, particularly in relation to GDP per capita (p < 0.01, q = 0.725), which serves as a crucial indicator for assessing regional economic conditions. The influence of SES on gastric cancer is believed to be mediated through its impact on well-established risk factors, such as health consciousness, Helicobacter pylori infection, dietary habits, and obesity. Individuals with a higher SES tend to exhibit greater health consciousness compared to those with lower economic standing, leading them to allocate more resources towards healthcare services, such as healthy examination and disease prevention. Adam B Weiner et al. found that prostate cancer diagnosis rates correlated positively with the S&P month close (p = 0.009, 95%CI 6.29–43.50); thus, economic hardship was closely related to decreased diagnosis rates of non-palpable prostate cancer [12].The association between Helicobacter pylori (Hp) infection and gastric cancer is widely acknowledged, with researchers suggesting that a disadvantaged SES and adverse childhood living conditions contribute to the acquisition of Hp infection [13]. SES also contributes to the migration of people's dietary patterns; in a cohort study, Silene Casari et al. discovered that urban individuals exhibit a more diverse dietary habit characterized by increased consumption of animal protein, simple sugars, and fibers compared to their rural counterparts. They proposed that urbanization the transition from rural to urban dietary habits in Africa [14]. Obesity is known as a risk factor for gastric cancer, especially for cardia gastric cancer [15, 16], research has found a closely correlation between obesity and different SES, G D Dinsa et al. found a positive correlation between SES and obesity in low-income countries or in countries with low human development index, the relationship might be mediated by eating off a large plate, eating at night and uncontrolled eating [17, 18]. In addition, the scope of health insurance coverage in Gansu has been expanding in recent years. This trend has led to an increased awareness of health among the population, which in turn has contributed to a rise in the diagnosis rates of gastric cancer within the region.

We observed that medical resource allocation played a crucial the incidence of gastric cancer in this study. We hypothesized that these factors, namely the density of medical institutions, hospital beds, and health technical staff per 10,000 individuals, might influence the diagnostic rate of gastric cancer rather than its occurrence. In the past years, particularly in rural regions, individuals residing in impoverished areas have tended to refrain from seeking medical attention at distant hospitals when experiencing discomfort and instead opted for silence. However, with the recent increase in balanced and abundant medical resources, local residents are now more easily able to The Geodetector analysis also revealed that diurnal temperature variation, regional rainfall, and ambient humidity significantly influenced the spatial distribution of gastric cancer in Gansu. However, there is currently no literature indicating a direct causal relationship between these climatic factors and tumorigenesis. Considering the geographic features of Gansu province, we hypothesized that these factors may indirectly impact the incidence of gastric cancer in this region.

Firstly, it can impact regional dietary patterns. Studies have suggested that unhealthy eating habits are closely linked to numerous diseases, particularly digestive cancers. Evidence has shown that the consumption of hot foods is associated with an elevated risk of esophageal cancer [19, 20]. High salt intake is a detrimental dietary practice that not only contributes to cardiovascular diseases but also exacerbates the risk of gastric cancer. Research has shown that excessive salt consumption can facilitate Hp colonization, induce proliferation and pit cell hyperplasia, cause glandular atrophy, and thus enhance the carcinogenic effects of cagA ( +) Hp strains while promoting Hp-associated carcinogenesis [21, 22], the crucial role of high salt intake in gastric cancer was also indicated in many cohort studies [23, 24]. Wuwei, situated in the northern part of Gansu province, serves as the principal city in the Hexi region with the highest incidence rate of gastric cancer within Gansu province. The arid climatic conditions prevalent in this area foster a proclivity for pickling habits, particularly among rural populations. According to various regional reports, over 95% of rural residents engage in vegetable pickling during winter months to diversify their dietary patterns; this practice typically lasts between two and four months annually. meanwhile, someone measured the level of nitrate, nitrite, N-dimethyl dinitramine and N-ethylnitrosamine, they revealed an increase in their levels with prolonged pickling time. Furthermore, individuals who regularly consumed pickled vegetables exhibited significantly higher levels of Nitrate and Nitrite in gastric juices compared to less frequent consumers. Therefore, we hypothesize that dry climatic conditions contribute to gastric cancer by promoting excessive salt consumption.

Secondly, crop styles may be impacted by climatic factors. Vitamins found in vegetables and fruits play a crucial role in human health, particularly vitamin C. It has been suggested that a deficiency of vitamin C is closely linked to various health issues such as cardiovascular diseases, blood disorders, and even cancer. In addition to its antioxidative effect on the human body, vitamin C has been shown to contribute to immune defense by supporting various cellular functions of both the innate and adaptive immune systems. Furthermore, it acts as a protective factor against many epithelial-derived cancers by destroying nitrification and inhibiting the accumulation of nitro compounds in the body [25, 26]. The vitamin C content in vegetables varies, with leafy vegetables having the highest concentration followed by root crops. A previous report indicates that cabbage, cauliflower, spinach, kale, and radish have significantly higher levels of vitamin C compared to cucumber, green onion, leek and potato. Due to varying climatic conditions across Gansu province's cities, vegetable types also differ. We take Wuwei and Longnan as examples. These two cities have the highest and lowest incidences of gastric cancer in Gansu province, respectively. It has been indicated that the vegetable types in Wuwei mainly consist of cucumbers, garlic shoots, peas, scallions, peppers, garlic, eggplant and bell peppers; while those in Longnan are primarily composed of garlic shoots, squash, garlic, cowpeas, kale, green beans lettuce spinach radishes and peas. According to the findings, it appears that the vitamin C content in vegetables grown in Wuwei is higher than that of Longnan. Regional vegetable species are closely linked to regional dietary patterns, particularly among rural residents who mostly rely on self-cultivated vegetables for supplementation. Therefore, regional crop styles may be a potential factor contributing to the spatial distribution of gastric cancer in Gansu province.

In addition, regional economic status can also be influenced by climatic conditions. Maximilian Kotz et al. assessed the economic output of 1,554 regions worldwide over the past four decades and discovered that increases in wet days and extreme daily rainfall led to a reduction in regional economic growth rates [27]. In this study, we have also observed a phenomenon where the economic status in arid regions (Wuwei, Zhangye, Jiayuguan and Jiuquan) surpasses that of humid regions (Longnan, Tianshui and Pingliang). As demonstrated by the correlation between economic status and gastric cancer above, we posit that climatic factors can influence the spatial distribution of gastric cancer through their impact on regional economic conditions.

Although we have found some interesting and significant findings in this study, there are still distinct limitations that need to be acknowledged. Numerous well-established risk factors, such as H. pylori infection, smoking, obesity, and dietary indicators (such as high salt/fat intake), significantly contribute to the development of gastric cancer. However, due to a lack of relevant data, we were unable to include these risk factors in our study. Furthermore, our analysis only yielded evidence of a close correlation between the some macro factors, such as SES, regional climate and medical resources, and the incidence of gastric cancer in Gansu. However, the underlying mechanisms behind this relationship remain elusive. Therefore, we intend to conduct Real World Studies (RWS) to gather further evidence to support our conclusions.

## Conclusion

Gastric cancer remains a significant threat and exhibits notable regional differences among the 14 cities in Gansu. We found the epidemiological characters and identified different risk clusters of gastric cancer in Gansu. Furthermore, we also revealed significant macro determinants of gastric cancer in Gansu, including SES, medical resource allocation and natural environmental conditions, of which, medical resource allocation and SES showed greater impact on gastric cancer.

## Data Availability

Datasets used and/or analyzed during the current study are available from the corresponding author on reasonable request.

## References

[1] Sung H, Ferlay J, Siegel RL, Laversanne M, Soerjomataram I, Jemal A, Bray F (2021). Global cancer statistics 2020: GLOBOCAN estimates of incidence and mortality worldwide for 36 cancers in 185 countries. CA Cancer J Clin.

[2] He Y, Wang Y, Luan F, Yu Z, Feng H, Chen B, Chen W (2021). Chinese and global burdens of gastric cancer from 1990 to 2019. Cancer Med.

[3] Lewandowska AM, Rudzki M, Rudzki S, Lewandowski T, Laskowska B (2019). Environmental risk factors for cancer—review paper. Ann Agric Environ Med.

[4] Sinha R, Caporaso N (1999). Diet, genetic susceptibility and human cancer etiology. J Nutr.

[5] Volanis D, Kadiyska T, Galanis A, Delakas D, Logotheti S, Zoumpourlis V (2010). Environmental factors and genetic susceptibility promote urinary bladder cancer. Toxicol Lett.

[6] Colao A, Muscogiuri G, Piscitelli P (2016). Environment and health: not only cancer. Int J Environ Res Public Health.

[7] Tueller G, Kerry R, Young SG (2023). Spatial investigation of the links between aflatoxins legislation, climate, and liver cancer at the global scale. Spat Spatiotemporal Epidemiol.

[8] Calderón-Gerstein WS, Torres-Samaniego G (2021). High altitude and cancer: an old controversy. Respir Physiol Neurobiol.

[9] Robinson TP (2000). Spatial statistics and geographical information systems in epidemiology and public health. Adv Parasitol.

[10] GBD 2019 Diseases and Injuries Collaborators. Global burden of 369 diseases and injuries in 204 countries and territories, 1990–2019: a systematic analysis for the Global Burden of Disease Study 2019. Lancet. 2020 Oct 17;396(10258):1204–1222. doi: 10.1016/S0140-6736(20)30925-9. 10.1016/S0140-6736(20)30925-9 PMC7567026 33069326

[11] Sifaki-Pistolla D, Chatzea VE, Frouzi E, Mechili EA, Pistolla G, Nikiforidis G, Georgoulias V, Lionis C, Tzanakis N (2022). Evidence-based conceptual collection of methods for spatial epidemiology and analysis to enhance cancer surveillance and public health. Int J Environ Res Public Health.

[12] Weiner AB, Conti RM, Eggener SE (2016). National economic conditions and patient insurance status predict prostate cancer diagnosis rates and management decisions. J Urol.

[13] Bures J, Kopácová M, Skodová Fendrichová M, Rejchrt S (2011). Epidemiologie helicobacter pylori. Vnitr Lek.

[14] Casari S, Di Paola M, Banci E, Diallo S, Scarallo L, Renzo S, Gori A, Renzi S, Paci M, de Mast Q, Pecht T, Derra K, Kaboré B, Tinto H, Cavalieri D, Lionetti P (2022). Changing dietary habits: the impact of urbanization and rising socio-economic status in families from burkina faso in Sub-Saharan Africa. Nutrients.

[15] Andrici J, Eslick GD (2015). Hot food and beverage consumption and the risk of esophageal cancer: a meta-analysis. Am J Prev Med.

[16] Tai WP, Nie GJ, Chen MJ, Yaz TY, Guli A, Wuxur A, Huang QQ, Lin ZG, Wu J (2017). Hot food and beverage consumption and the risk of esophageal squamous cell carcinoma: a case-control study in a northwest area in China. Medicine (Baltimore).

[17] Gaddy JA, Radin JN, Loh JT, Zhang F, Washington MK, Peek RM, Algood HM, Cover TL (2013). High dietary salt intake exacerbates Helicobacter pylori-induced gastric carcinogenesis. Infect Immun.

[18] Thrift AP, Wenker TN, El-Serag HB (2023). Global burden of gastric cancer: epidemiological trends, risk factors, screening and prevention. Nat Rev Clin Oncol.

[19] Song M, Choi JY, Yang JJ, Sung H, Lee Y, Lee HW, Kong SH, Lee HJ, Kim HH, Kim SG, Yang HK, Kang D (2015). Obesity at adolescence and gastric cancer risk. Cancer Causes Control.

[20] Dinsa GD, Goryakin Y, Fumagalli E, Suhrcke M (2012). Obesity and socioeconomic status in developing countries: a systematic review. Obes Rev.

[21] Pigeyre M, Rousseaux J, Trouiller P, Dumont J, Goumidi L, Bonte D, Dumont MP, Chmielewski A, Duhamel A, Amouyel P, Dallongeville J, Romon M, Meirhaeghe A (2016). How obesity relates to socio-economic status: identification of eating behavior mediators. Int J Obes (Lond).

[22] Fox JG, Dangler CA, Taylor NS, King A, Koh TJ, Wang TC (1999). High-salt diet induces gastric epithelial hyperplasia and parietal cell loss, and enhances Helicobacter pylori colonization in C57BL/6 mice. Cancer Res.

[23] Lin SH, Li YH, Leung K, Huang CY, Wang XR (2014). Salt processed food and gastric cancer in a Chinese population. Asian Pac J Cancer Prev.

[24] Lazarevic K, Nagorni A, Rancic N, Milutinovic S, Stosic L, Ilijev I (2010). Dietary factors and gastric cancer risk: hospital-based case control study. J BUON.

[25] Carr AC, Maggini S (2017). Vitamin C and immune function. Nutrients.

[26] Bartsch H, Frank N (1996). Blocking the endogenous formation of N-nitroso compounds and related carcinogens. IARC Sci Publ.

[27] Kotz M, Levermann A, Wenz L (2022). The effect of rainfall changes on economic production. Nature.

